# The prevalence of diabetes distress among patients with type 2 diabetes in Jordan

**DOI:** 10.1016/j.jtumed.2023.04.002

**Published:** 2023-04-15

**Authors:** Duaa A. Hiasat, Maryam B. Salih, Aseel H. Abu Jaber, Obada F. Abubaker, Yousef A. Qandeel, Bushra A. Saleem, Sally I. Aburumman, Abdel Rahman H. Al-Sayyed, Tariq I. Hussein, Dana Hyassat

**Affiliations:** a Faculty of Medicine, Al-Balqa' Applied University, Jordan; b National Center for Diabetes, Endocrinology and Genetics, Jordan

**Keywords:** ضائقة السكري, مرض السكري, الأردن, انتشار, السكري من النوع 2, Diabetes distress, Diabetes mellitus, Jordan, Prevalence, Type 2 diabetes

## Abstract

**Objectives:**

Diabetes distress (DD) is a state of emotional distress that evolves from living with chronic disease and the burden of daily adjustments of medications and lifestyle. This study investigated the prevalence of DD in patients with type 2 diabetes mellitus (T2DM) in Jordan and the related sociodemographic and medical factors.

**Methods:**

We conducted a cross-sectional study in 608 patients with T2DM in Jordan, ranging from ages 15 to 80 years. The participants filled out a questionnaire where they were asked to self-assess their DD using the Diabetes Distress Scale. In all, 32 participants were excluded according to the exclusion criteria, which resulted in 576 people being included in this study.

**Results:**

The overall prevalence of DD was 53% (25% had moderate distress and 28% had high distress). Emotional distress had the highest prevalence among the DD subscales, with a total prevalence of 58.8%. The data showed a significant association of DD with different factors including age, the presence of diabetic complications, the type of medication used, and medication adherence.

**Conclusion:**

This study showed a high prevalence of DD (53%). This finding should raise awareness to healthcare providers about the importance of screening for DD as part of the treatment guidelines, especially in patients who are on multiple medication regimens for DM; patients who have previous medical complications related to DM; and those who exhibit poor adherence to medications, which was found to be a risk factor of DD in this study.

## المخلص

**أهداف البحث:**

ضائقة داء السكري هي حالة من الضائقة العاطفية التي تنشأ من العيش مع مرض مزمن وعبء التعديلات اليومية للأدوية ونمط الحياة. تدرس هذه الدراسة انتشار ضائقة داء السكري في مرضى السكري من النوع 2 في الأردن والعوامل الاجتماعية والديموغرافية والطبية.

**طرق البحث:**

استخدمنا دراسة مستعرضة مع حجم عينة يبلغ 608 مرضى سكري من النوع 2 في الأردن، بين سن 15-80 عاما. قام المشاركون بتعبئة استبيان حيث طلب منهم تقييم مقياس ضائقة داء السكري الخاص بهم. تم استبعاد 32 مشاركا وفقا لمعايير الاستبعاد، مما يؤدي إلى إدراج 576 شخصا في هذه الدراسة.

**النتائج:**

إن انتشار ضائقة داء السكري الإجمالي هو 53٪ (25٪ كمرض معتدل و 28٪ كمرض عالي)، والضائقة العاطفية هي الأعلى انتشاراً بين المقاييس الفرعية لضائقة السكري بانتشار إجمالي يبلغ 58.8٪. تظهر البيانات وجود علاقة كبيرة بين ضائقة السكري وعوامل مختلفة بما في ذلك العمر، وجود مضاعفات السكري، نوع الدواء المستخدم، والالتزام بالدواء.

**الاستنتاجات:**

أظهرت هذه الدراسة انتشارا عاليا لضائقة داء السكري (53٪) ويجب أن يساعد ذلك مقدمي الرعاية الصحية على معرفة أهمية فحص ضائقة السكري كجزء من إرشادات العلاج، خاصة في المرضى الذين يتناولون عدة أنواع من الأدوية للسكري، والمرضى الذين لديهم مضاعفات طبية سابقة متعلقة بالسكري والمرضى الذين يظهرون انخفاضا في الالتزام بالأدوية؛ وجد أنهم معرضون لخطر الإصابة بمرض السكري في هذه الدراسة.

## Introduction

Diabetes mellitus (DM) is one of the most common chronic diseases and has a critical impact on a patient's life. In addition to the complex self-management and medical treatments for DM, the disease has a significant effect on a patient's lifestyle and relationships.^1^ In most cases, diabetic patients and their families face many challenges in balancing DM management and a normal lifestyle, which has a major impact on the patient's psychology, well-being, and quality of life (QoL).

One of the psychological effects that diabetic patients may experience is diabetes distress (DD), which is defined as a state of emotional distress that results from living with DM as a chronic disease and the need for continuous self-management and its effect on the patient's mental health.^2^ DD not only affects a patient's psychology but also affects a patient's health and is associated with poor health outcomes.^3^

In Jordan, according to the National Center for Diabetes, Endocrinology, and Genetics, the prevalence of DM and pre-DM is about 45%.^4^ Despite the high prevalence, there have been few studies on the psychosocial impact of DM on a patient's life and DD. In addition, most of the guidelines on DM care have focused on the medical aspects of initial management without addressing the psychological needs of the patients.

DD is a common worldwide problem in patients with both type 1 DM (T1DM) and T2DM in different age groups. In 2001, a study was conducted to study the effects of psychological barriers on DM management and improvement (DAWN 1).^5^ This study was carried out in multiple countries, and showed that about 41% of diabetic patients suffered from psychological problems related to DM and distress associated with disease management. Furthermore, in the DAWN 2 study that was conducted in 2013, DD was reported by 44.6% of the participants.^6^

Multiple studies were conducted in the Middle East in 2020 to study the prevalence of DD among patients with T2DM and to assess the associated factors. For example, cross-sectional studies conducted in Qatar^7^ and KSA^8^ showed a prevalence of DD of 40.3% and 35.5%, respectively.

Another study conducted in Kuwait^9^ showed a prevalence of 14%. In terms of developing countries, a cross-sectional study conducted in Iran^10^ in 2018 found that the prevalence was 48.6%. However, a systematic review and meta-analysis^11^ involving 55 papers from different nations found an overall DD prevalence of 36%.

The differences in prevalence of DD might result from the differences in assessment tools. Two scales are used to assess DD: the Problem Area in Diabetes (PAID) scale and the Diabetes Distress scale (DDS).^12^ Both scales were validated and confirmed to have good psychometric properties, but there are differences in their content and psychometric characteristics. The DDS focuses more on behavioral problems regarding DM self-management and physician-related distress. On the other hand, the PAID scale focuses on emotional concerns, problems related to diet, and their complications. Those main differences in content make the DDS strongly associated with management and metabolic outcome, whereas the PAID scale is associated with QoL and psychological well-being.^13^

According to different studies, multiple factors are related to DD and can be employed to predict the patients who may have DD. Those factors are a combination of sociodemographic and medical variables such as a specific age group, female gender, experiencing one or more DM complications, having a longer disease duration, injections as a type of medication, high body mass index (BMI), and a sedentary lifestyle in general.^14^

Clinically and as expected, DD is considered a serious condition due to its effects – as a psychological barrier – on patient's self-management and self-neglect, and subsequently on glycemic control and glycated hemoglobin (HbA1c) levels. Few studies have assessed the association of DD with glycemic control. A recent study in KSA^15^ showed an association between DD and adherence to medication and subsequently glycemic control. Other studies have assessed the association between DD and glycemic control compared to depression, and studies conducted in Japan^16^ and the Netherlands^17^ reported that DD is more strongly associated with glycemic control than depressive symptoms. However, a few studies did not find this association, one of which was a cohort study at the primary care level, which unexpectedly, reported that neither depressive symptoms nor DD are associated with worsening glycemic control in patients diagnosed early with DM.^18^

According to the World Health Organization, QoL is defined as “a person's perception of his position in life in the context of the culture and value systems in which he lives and concerning his goals, expectations, standards, and concerns”.^19^ A few studies have focused mainly on the impact of DD on QoL. One of those studies concluded that DD has a negative association with health-related QoL, and the association is more significant in diabetics patients who do not have supportive family or friends, as well as in patients with multiple comorbidities.^20^

## Materials and Methods

### Study design

We conducted a cross-sectional study to assess the prevalence and associated factors of DD in patients with T2DM in Jordan. This study was conducted between December 2021 and February 2022 at the National Center for Diabetes Endocrinology and Genetics and Al-Salt New Hospital (Jordan), as they are well-known centers serving high numbers of diabetic patients from different age groups, disease stages, and backgrounds.

### Study population

This study included 608 patients with T2DM who were able to provide informed consent and communicate. The study included both males and females between the ages of 15 and 80 years old. The exclusion criteria were patients with a current or previous psychiatric history (previous psychiatric visit or history of using psychiatric medication), patients with cancer, hospitalized patients, or patients who experienced unpleasant events during the data collection period. Overall, 32 patients were excluded from the study.

### Sampling technique

The study population was defined in the previous section. The Epicalc 2000 calculator was used to calculate the sample size and estimate the prevalence according to studies that have been conducted in the Middle East, using a significance of 0.05% and a power of 80%.

Proportion: 35.00%
Null hypothesis value: 30.00%
Significance: 0.05
Power: 80%
Sample size: 674

A systemic sampling technique was used, a questionnaire was given to every second patient who came to the outpatient diabetic clinic, and the first patient was the starting point. The same systemic sampling was done for each diabetic clinic. The data were collected from 608 participants and 32 patients were excluded, yielding a total of 576 participants included in the study.

### Data collection and variables

The data were collected using a self-reported questionnaire, which was divided into three parts.

#### Sociodemographic information

The variables of age, gender, education level, marital status, and occupation were collected using a sociodemographic questionnaire.

#### Clinical information

The clinical information included clinical variables such as type of DM, type of management (insulin injection, oral medication, or both), adherence to medication, existence of another co-morbid disease, and chronic medication use.

#### DD

The DDS was used to assess and measure the degree of patient distress.^21^ The Arabic version of the DDS was used, which has been approved and validated.^22^ The DDS includes 17 items related to distress, and the patients evaluated the degree of distress for each item in a range between 1 and 6, where grade 1 means no problem and 6 means very serious problems.

The DDS score was calculated by measuring the average score of the 17 items. The minimum score is 1, and the maximum score for this scale is 6. The score is classified according to severity into three levels of distress: <2.0 = no or mild distress (not significant), 2.0–2.9 = moderate distress, and ≥3.0 = severe distress.^23^

The DDS measures four dimensions of distress: emotional burden, physician-related distress, regimen-related distress, and interpersonal distress.

### Data analysis

Data were translated and transferred to a computerized database. Statistical analyses were conducted using IBM SPSS Statistics version 26 (IBM, Armonk, NY, USA) in conjunction with Microsoft Excel. Descriptive statistics are reported as frequency, percentage, mean and standard deviations. The association between the level of DD and sociodemographic and clinical variables was estimated using the chi-square test and one-way analysis of variance (ANOVA), and the significance level was defined as *P* < 0.05.

## Results

### Participants

A total of 576 patients with T2DM were included in the study: 319 (55.4%) females and 257 (44.6%) males, with a mean age of 56.65 ± 10.26 years. Table 1 shows the detailed sociodemographic and clinical characteristics of the participants.

**Table 1:** Sociodemographic and clinical characteristics of the participants.

Characteristic	Frequency/mean ± SD	Percent
Age	56.65 ± 10.266	
≤50	143	24.8%
>50	433	75.2%
Sex		
Female	319	55.4%
Male	257	44.6%
Marital status		
Married	473	82.1%
Single	16	2.8%
Divorced	14	2.4%
Widow	73	12.7%
Education level		
School	301	52.2%
Diploma	100	17.4%
Bachelor's	139	24.1%
Master and above	36	6.3%
Occupation status		
Office work	91	15.8%
On the field	89	15.5%
Unemployed	396	68.8%
Type of medication		
Insulin	66	11.5%
Oral	357	62%
Oral + insulin	153	26.6%
Presence of complications related to diabetes		
Yes	181	31.4%
No	395	68.8%
Adherence to medication		
Yes	528	91.7%
No	48	8.3%
Presence of comorbidity		
Yes	395	68.8%
No	181	31.4%
Chronic drug use		
Yes	420	72.9%
No	156	27.1%

### Prevalence of DD

The mean score for total DD was 1.8 ± 0.84. Emotional distress had the highest score of 2.0 ± 0.9 followed by regimen-related distress with a score of 1.78 ± 0.88. Interpersonal distress and physician-related distress both had the same score of 1.61 ± 0.84.

The total prevalence of DD was 53% (25% had moderate distress and 28% had high distress). Emotional distress had the highest prevalence among the DD subscales with a total prevalence of 58.8% (17.7% had moderate distress and 41.1% had high distress), followed by regimen-related distress (prevalence of 47.5%). The prevalence of physician-related distress and interpersonal distress was 37.7% and 37.1%, respectively (Table 2).

**Figure:**
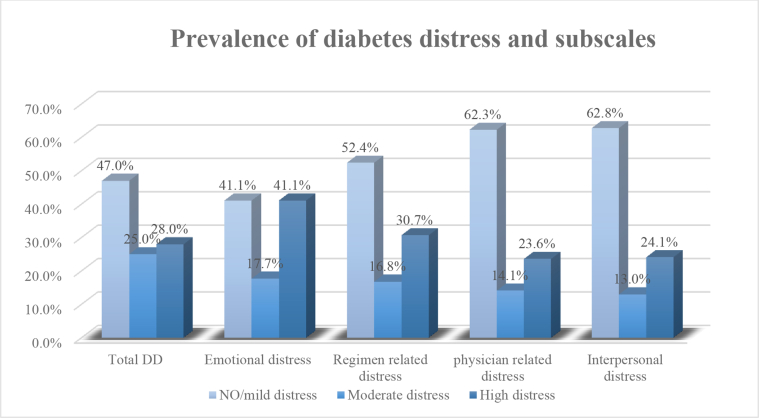

**Table 2:** Prevalence of diabetes distress and subscales.

Type of distress	No/mild distress	Moderate distress	High distress
Total diabetes distress	47%	25%	28%
Emotional-related distress	41.1%	17.7%	41.1%
Physician-related distress	62.3%	14.1%	23.6%
Regimen-related distress	52.4%	16.8%	30.7%
Interpersonal-related distress	62.8%	13.0%	24.1%

### Association

The total degree of distress score was substantially linked to age (*P =* 0.001), diabetic complications (*P* = 0.000), medication type (*P* = 0.05), and medication adherence (*P* = 0.012). Sex, marital status, educational level, occupation, and the existence of comorbidities had no significant association with the degree of distress (Table 3).

**Table 3:** Sociodemographic and clinical variables according to levels of distress.

Characteristic	No distress	Moderate distress	High distress	*P* value
Age				
≤50	51 (35%)	36 (25%)	56 (40%)	
>50	220 (51%)	108 (25%)	105 (24%)	0.001∗
Sex				
Female	150 (47%)	73 (23%)	96 (30%)	0.606
Male	121 (47%)	71 (28%)	65 (25%)	
Marital status				
Married	221 (47%)	117 (25%)	135 (28%)	
Single	4 (25%)	5 (31%)	7 (44%)	0.216
Divorced	5 (36%)	6 (43%)	3 (21%)	
Widow	41 (56%)	16 (22%)	16 (22%)	
Education level				
School	144 (48%)	71 (24%)	86 (28%)	
Diploma	51 (51%)	20 (20%)	29 (29%)	0.439
Bachelor's	60 (43%)	39 (28%)	40 (29%)	
Master and above	16 (44%)	14 (39%)	6 (17%)	
Occupation status				
Office work	37 (41%)	22 (24%)	32 (35%)	
On the field	38 (43%)	22 (25%)	29 (32%)	0.277
Unemployed	196 (50%)	100 (25%)	100 (25%)	
Type of medication				
Insulin	27 (41%) 18	(27%) 21	(32%)	
Oral	186 (52%)	85 (24%)	86 (24%)	0.027∗
Oral + insulin	58 (38%)	41 (27%)	54 (35%)	
Presence of complications related to diabetes				
Yes	65 (36%)	47 (26%)	69 (38%)	0.000∗
No	206 (52%)	97 (25%)	92 (23%)	
Adherence to medication				
Yes	258 (49%)	129 (24%)	141 (27%)	0.012∗
No	13 (27%)	15 (31%)	20 (42%)	
Presence of comorbidity				
Yes	185 (47%)	100 (25%)	110 (28%)	
No	86 (48%)	44 (24%)	51 (28%)	0.967
Chronic drug use				
Yes	195 (46%)	105 (25%)	120 (29%)	
No	76 (49%)	39 (25%)	41 (26%)	0.844

∗Statistically significant difference (*P* < 0.05)

In terms of the DD subscale, emotional-related and physician-related distress were also significantly correlated with occupational status (*P* = 0.047 and 0.030, respectively). However, regimen-related distress and interpersonal-related distress only had a significant relationship with medication adherence (*P* = 0.001 and 0.033, respectively).

## Discussion

In our study, the total prevalence of DD was about 53% for moderate to high DD, which is higher than the prevalence reported in other countries such as KSA (25%),^24^ Qatar (40.3%),^7^ Malaysia (49.2%),^25^ United States (51.3%),^26^ Bangladesh (48.5%),^27^ China (43%),^28^ and Canada (39%),^29^ all of which also used the DDS-17 scale for DD assessment. However, two studies from Germany used the PAID questionnaire and found a DD prevalence of 8.9%^30^ and 10.7%.^31^

This study showed that emotional-related distress had the highest prevalence among the DD subscales, followed by regimen-, physician-, and interpersonal-related distress. The same order of distress was found in the KSA^8^ study, except that regimen-related distress was higher than emotional distress. Studies in Qatar^7^ and KSA^8^ showed that emotional- and regimen-related distress had the highest prevalence among the subclasses of DD. This explains the importance of patients' emotions and the need to treat their concerns regarding how to deal with DM including medication management.

This study found that the prevalence of DD was equal in both genders however, females had a higher level of emotional-related distress than males. Females usually report higher levels of psychological distress than males, possibly because they are more expressive, face more stressors, lack coping resources or mechanisms, and have different biological responses to depression.^8^

The majority of participants in the current study were >50 years old (75.2%), with a total DD prevalence of 49% compared to those ≤50 years old, who had a total DD prevalence of 65%. Similar to this study, some literature has shown that younger adults are at higher risk of DD,^32^ possibly because they may have more reactions to stressors and less ability to deal with stress,^33^ unlike older adults who may have developed better coping mechanisms.

According to the type of medication, those on just oral medications have a lower DD prevalence, compared to those on insulin or both oral and insulin. Insulin injections have also been associated with DD in other studies.^8^ The need for injections may be related to poor control of blood glucose or worsening of the diabetic patient's health, resulting in increased distress. DD may also be related to side effects from the injection such as pain, fear of hypoglycemia, and difficulty in administering the insulin injection. These factors may underlie the association between insulin and DD.

According to previous literature, there is a strong relationship between DD and poor control of HbA1c.^15^ Thus, regarding the association between DM control and adherence to medications with DD, in this study, the prevalence of total DD was high in non-adherent participants compared to those with medication adherence. Therefore, DM self-management education has a significant role in improving DD, which leads to improvement in glycemic control.^34^

The literature has shown a strong association between DD and complications related to DM.^35^ In our study, we found that participants with DM-related complications had a higher prevalence of DD compared to those without DM-related complications.

Regarding emotional- and physician-related distress, we found another significant factor that specifically affected these two subclasses, namely, occupational status, specifically office work. The prevalence of emotional- and physician-related distress among office workers was 67% and 49% respectively, which was lower than that in unemployed patients (57% and 45%, respectively).

In brief, more attention should be paid to DD, as it is as common in Jordan as well as in other countries. Therefore, we should focus on patients who have risk factors for DD such as female gender, patients taking insulin injections, those complaining of complications related to DM, and patients who have problems with medication adherence. Identifying patients who are most likely to suffer from DD will help in the early diagnosis of this condition and provide patients the care and attention they need, either psychologically or medically, in order to improve their QoL, mental health, and overall well-being.

### Strength and limitations

This was a qualitative study, which means that it was objective, fair, and unbiased. One of the important strengths of this study was that informed consent was provided by all participants and their identity remained anonymous. The most important limitation of this study was that it only included secondary and tertiary care levels; thus, patients who mostly received primary levels of care were not included.

## Conclusion

In conclusion, this study showed a higher prevalence of DD in Jordan (53%) compared with other countries in the Middle East and worldwide. Younger age, a combination of oral and injectable medications, experiencing DM complications, and poor medication adherence were associated with DD in our study. Therefore, early screening for DD in those patients may help with a management plan and early intervention to improve QoL for diabetic patients and provide the necessary training to patients and their families for managing DD.

## Source of funding

This research did not receive any specific grant from funding agencies in the public, commercial, or not-for-profit sectors.

## Conflict of interest

The authors have no conflict of interest to declare.

## Ethical approval

Regarding ethical considerations, written informed consent was provided by all participants, and the study was approved by ethics committee of the Faculty of Medicine at Al-Balqa Applied University. The study was also licensed by the National Institute for Diabetes, Endocrinology, and Genetics through the institutional review board on December 28, 2021.

## Authors contributions

DAH was the main supervisor of the study and contributed its design and implementation. AAJ, MBS, OFA, YAQ, BAS, and SIA designed and directed the research; collected, organized, and analyzed the data; and wrote the initial, second, and final drafts of the manuscript. AAS and TIH contributed to the data collection. DH contributed to the data collection from National Institute for Diabetes, Endocrinology, and Genetics. All authors have critically reviewed and approved the final draft and are responsible for the content and similarity index of the manuscript.

## References

[1] Nash J. (2014). Understanding the psychological impact of diabetes and the role of clinical psychology. J Diabetes Nurs.

[2] Mascott C. (2014). Diabetes distress. Diabetes Self Manag.

[3] Nicolucci A., Rossi M.C., Pellegrini F., Lucisano G., Pintaudi B., Gentile S. (2014). Benchmarking network for clinical and humanistic outcomes in diabetes (BENCH-D) study: protocol, tools, and population. SpringerPlus.

[4] المركز الوطني للسكري والغدد الصم والوراثة | الموقع الرسمي [Internet]. [cited 2022 Oct 4]. Available from: https://ncd.org.jo/.

[5] Peyrot M., Rubin R.R., Lauritzen T., Snoek F.J., Matthews D.R., Skovlund S.E. (2005). Psychosocial problems and barriers to improved diabetes management: results of the cross-National Diabetes Attitudes, Wishes and Needs (DAWN) study. Diabet Med.

[6] Peyrot M., Burns K.K., Davies M., Forbes A., Hermanns N., Holt R. (2013). Diabetes attitudes Wishes and Needs 2 (DAWN2): a multinational, multi-stakeholder study of psychosocial issues in diabetes and person-centred diabetes care. Diabetes Res Clin Pract.

[7] Abdalla H., Alnuaimi A., Gadallah A. (2020). Prevalence of diabetes distress among people with type 2 diabetes at primary health care in Qatar: a cross – sectional study. World Fam Med J.

[8] AlOtaibi AA, Almesned M, Alahaideb TM, Almasari SM, Alsuwayt SS (2021 Sep). Assessment of diabetes-related distress among type 2 diabetic patients, Riyadh, Saudi Arabia. J Fam Med Prim Care.

[9] Al-Ozairi E., Al Ozairi A., Blythe C., Taghadom E., Ismail K. (2020). The epidemiology of depression and diabetes distress in type 2 diabetes in Kuwait. J Diabetes Res.

[10] Azadbakht M., Taheri Tanjani P., Fadayevatan R., Froughan M., Zanjari N. (2020). The prevalence and predictors of diabetes distress in elderly with type 2 diabetes mellitus. Diabetes Res Clin Pract.

[11] Perrin N.E., Davies M.J., Robertson N., Snoek F.J., Khunti K. (2017). The prevalence of diabetes-specific emotional distress in people with Type 2 diabetes: a systematic review and meta-analysis. Diabet Med.

[12] Dennick K., Sturt J., Speight J. (2017). What is diabetes distress and how can we measure it? A narrative review and conceptual model. J Diabetes Complicat.

[13] Schmitt A., Reimer A., Kulzer B., Haak T., Ehrmann D., Hermanns N. (2016). How to assess diabetes distress: comparison of the problem areas in diabetes scale (PAID) and the diabetes distress scale (DDS). Diabet Med.

[14] Fisher L., Mullan J.T., Skaff M.M., Glasgow R.E., Arean P., Hessler D. (2009). Predicting diabetes distress in patients with Type 2 diabetes: a longitudinal study. Diabet Med.

[15] Fayed A., AlRadini F., Alzuhairi R.M., Aljuhani A.E., Alrashid H.R., Alwazae M.M. (2022). Relation between diabetes related distress and glycemic control: the mediating effect of adherence to treatment. Prim Care Diabetes.

[16] Tsujii S., Hayashino Y., Ishii H. (2012 Nov 1). Diabetes distress, but not depressive symptoms, is associated with glycaemic control among Japanese patients with Type 2 diabetes: diabetes Distress and Care Registry at Tenri (DDCRT 1). Diabet Med.

[17] Van Bastelaar K.M.P., Pouwer F., Geelhoed-Duijvestijn P.H.L.M., Tack C.J., Bazelmans E., Beekman A.T. (2010 Jul 1). Diabetes-specific emotional distress mediates the association between depressive symptoms and glycaemic control in Type 1 and Type 2 diabetes. Diabet Med.

[18] Ismail K., Moulton C.D., Winkley K., Pickup J.C., Thomas S.M., Sherwood R.A. (2017). The association of depressive symptoms and diabetes distress with glycaemic control and diabetes complications over 2 years in newly diagnosed type 2 diabetes: a prospective cohort study. Diabetologia.

[19] WHOQOL - Measuring Quality of Life| The World Health Organization [Internet]. [cited 2022 Oct 4]. Available from: https://www.who.int/tools/whoqol.

[20] Chew B.H., Mohd-Sidik S., Shariff-Ghazali S. (2015). Negative effects of diabetes-related distress on health-related quality of life: an evaluation among the adult patients with type 2 diabetes mellitus in three primary healthcare clinics in Malaysia. Health Qual Life Outcomes.

[21] Polonsky W.H., Fisher L., Earles J., Dudl R.J., Lees J., Mullan J., Jackson R.A. (2005). Assessing psychosocial distress in diabetes. Diabetes Care.

[22] Darawad M.W., Hammad S., Samarkandi O.A., Hamdan-Mansour A.M., Khalil A.A. (2017). Evaluating the psychometric properties of the Arabic version of the diabetes distress scale. J Psychosoc Nurs Ment Health Serv.

[23] Fisher L., Hessler D.M., Polonsky W.H., Mullan J. (2012). When is diabetes distress clinically meaningful? Establishing cut points for the diabetes distress scale. Diabetes Care.

[24] Aljuaid M.O., Almutairi A.M., Assiri M.A., Almalki D.M., Alswat K. (2018). Diabetes-related distress assessment among type 2 diabetes patients. J Diabetes Res.

[25] Chew B.H., Vos R., Mohd-Sidik S., Rutten G.E.H.M. (2016). Diabetes-related distress, depression and distress-depression among adults with type 2 diabetes mellitus in Malaysia. PLoS One.

[26] Fisher L., Glasgow R.E., Strycker L.A. (2010). The relationship between diabetes distress and clinical depression with glycemic control among patients with type 2 diabetes. Diabetes Care.

[27] Islam M., Karim M., Habib S., Yesmin K. (2013). Diabetes distress among type 2 diabetic patients. Int J Med Biomed Res.

[28] Hu Y., Li L., Zhang J. (2020). Diabetes distress in young adults with type 2 diabetes: a cross-sectional survey in China. J Diabetes Res.

[29] Wong E.M., Afshar R., Qian H., Zhang M., Elliott T.G., Tang T.S. (2017). Diabetes distress, depression and glycemic control in a Canadian-based specialty care setting. Can J Diabetes.

[30] Kuniss N., Kramer G., Müller N., Kloos C., Lehmann T., Lorkowski S. (2016 May). Diabetes-related burden and distress is low in people with diabetes at outpatient tertiary care level. Exp Clin Endocrinol Diabetes.

[31] Kuniss N., Rechtacek T., Kloos C., Müller U.A., Roth J., Burghardt K. (2017). Diabetes-related burden and distress in people with diabetes mellitus at primary care level in Germany. Acta Diabetol.

[32] Stoop C.H., Nefs G., Pop V.J., Wijnands-van Gent C.J.M., Tack C.J., Geelhoed-Duijvestijn P.H.L.M. (2014). Diabetes-specific emotional distress in people with Type 2 diabetes: a comparison between primary and secondary care. Diabet Med.

[33] Schieman S., Van Gundy K., Taylor J. (2002). The relationship between age and depressive symptoms: a test of competing explanatory and suppression influences. J Aging Health.

[34] Lin K., Park C., Li M., Wang X., Li X., Li W. (2017 Sep 1). Effects of depression, diabetes distress, diabetes self-efficacy, and diabetes self-management on glycemic control among Chinese population with type 2 diabetes mellitus. Diabetes Res Clin Pract.

[35] Khashayar P., Shirzad N., Zarbini A., Esteghamati A., Hemmatabadi M., Sharafi E. (2022). Diabetes-related distress and its association with the complications of diabetes in Iran. J Diabetes Metab Disord.

